# ‘The Letter Says I May or May Not Be Eligible… It Is a Big Doubt and Frustrating:’ A Qualitative Study on Barriers and Facilitators to Children's Oral Healthcare From the Perspective of Karen Refugee Parents in Victoria

**DOI:** 10.1111/hex.70110

**Published:** 2024-11-19

**Authors:** Sudheer Babu Balla, Jyothi Tadakamadla, Santosh Kumar Tadakamadla

**Affiliations:** ^1^ Dentistry and Oral Health Department of Rural Clinical Science La Trobe Rural Health School La Trobe University Bendigo Victoria Australia

**Keywords:** access, children, Karen Refugee Community, oral healthcare, parents, qualitative research

## Abstract

**Background:**

Australia has a longstanding tradition of resettling refugees and individuals in humanitarian need. Among these, the Karen community from Southeast Asia is rapidly growing in Australia. The absence of data on the barriers they face in accessing dental services is concerning. This study explores the barriers and facilitators Karen refugees encounter when seeking oral healthcare for their children in Australia, aiming to understand their experiences.

**Methods:**

Using a qualitative research design with a phenomenological approach, we conducted semi‐structured interviews with 23 parents (17 females and 6 males) who had been in Australia for 1–17 years. Each interview, lasting between 35 and 60 min, was audio‐recorded and transcribed verbatim. The transcripts were thematically analysed through an inductive, data‐driven approach, focusing on open coding and participant‐based meanings.

**Findings:**

Nine main themes were identified. At the individual level, cultural practices, parental behaviours and perceptions were the primary barriers. At the organisational level, long waiting lists in the public dental system were significant barriers. Additionally, a lack of knowledge about financial benefits and government support for children's dental care deterred refugees from seeking dental services. The results also highlighted the strengths of support networks, free dental care for children and school‐based dental care programmes. Parents reported experiences of inadequate oral healthcare, citing issues such as insufficient cultural sensitivity training among dental service providers, interpreter problems and shortages. These experiences revealed gaps in the provision of oral healthcare services.

**Conclusion:**

When designing tailored oral health promotion programs, all stakeholders must consider the lived experiences of refugees as valuable sources of information.

**Patient or Public Contribution:**

The authors thank the parents and carers from the Karen refugee community for sharing their experiences with the oral healthcare of their children. Recruitment was facilitated by the Karen Organisation of Bendigo and Bendigo Community Health Services. An interpreter from the Karen refugee community assisted in all the interviews.

## 1 Introduction

Throughout human history, many people have been forced to leave their homes and countries because of persecution, conflict, violence and violations of their human rights. According to the United Nations Refugee Agency (UNHCR) report, about 108.4 million people worldwide have had to flee [1]. Among them, nearly 35.3 million are refugees and asylum seekers, who are a priority group for UNHCR. A refugee, as defined by the UNHCR convention, is someone who has a ‘well‐founded fear of being persecuted for reasons of race, religion, nationality, membership of a particular social group or political opinion, is outside the country of his nationality and is unable or, owing to such fear, is unwilling to avail himself of the protection of that country’ [2]. On the other hand, an asylum seeker is waiting for their refugee status to be determined [3].

Australia has a long history of welcoming refugees and others needing humanitarian assistance [4]. It is one of the few countries with a permanent programme for resettling people. Australian government statistics show that from 1947 to 2022, 940,159 refugees and humanitarian entrants have arrived in Australia [5]. In the 2021–22 period, 13,307 resettlement visas were given out. Among these, 11,545 were offshore humanitarian visas (62.7% for refugees and 37.3% for special humanitarian categories), and 1,762 were permanent Protection visas [6]. The majority of these visas, around 62.3%, were given to people from Asia (such as Afghans, Myanmar nationals, Iraqis and Syrians), 30.1% to people from the Middle East, 7% to those from Africa and less than 1% to individuals from the Americas [6].

Myanmar, formerly known as Burma, boasts significant ethnic and cultural diversity [7]. Among its diverse ethnic groups is the Karen ethnic group. Following the British departure from the region in 1948, the Burmese government persecuted the people from ethnic minority groups, leading to their forced displacement into camps across the border in Thailand [8]. Many of these camps have existed for over 40 years [9]. Since 2005, the UN has been resettling camp residents, leading Burmese ethnic minorities, including Karen refugees, to seek sanctuary in countries like Australia, Canada and the United States [10]. Australia continues to prioritise resettlement for people from Myanmar. Bendigo, a regional city in central Victoria, has been resettling Karen refugees since 2007, making them one of the largest refugee communities in Victoria over the past 15 years [11, 12].

The existing body of literature widely acknowledges the various factors influencing the oral health of refugees. These factors encompass post‐migration stress, resettlement challenges and a range of barriers, including language, cultural differences, social dynamics, structural obstacles and financial constraints [13, 14, 15, 16]. Furthermore, access to oral healthcare for this population is impacted by the healthcare systems of host countries, which is variable between the nations [17]. In Australia, despite the entitlement of humanitarian migrants, including refugees, to universal healthcare, dental services for most populations are notably excluded from coverage by the Commonwealth government [18]. Moreover, Australia's predominantly private oral healthcare sector, where approximately 85% of the workforce operates, presents additional challenges to accessing oral healthcare services, even for socio‐economically disadvantaged Australians [19]. In response to the recognised vulnerability of these groups, Dental Health Services Victoria (DHSV) has expanded eligibility for public dental services to encompass humanitarian migrants, including refugees [20]. However, despite these efforts, the utilisation of public dental services among refugees remains disproportionately low [21].

In 2014, the Australian Commonwealth government implemented the Child Dental Benefits Schedule (CDBS), a policy aimed at providing dental funding for children. Administered through Medicare, it allows children aged 0–17 to access up to $1095 in benefits over 2 years for basic dental services [22, 23, 24]. Despite the intended purpose of promoting good oral health among children, the overall utilisation of this policy has remained low [25]. Evidence regarding access to CDBS by children from priority populations indicates that only 8% of low‐income households utilise dental services through this scheme [26]. However, the extent of CDBS utilisation by children from refugee backgrounds remains unclear. Given the vulnerability of the Karen refugee population to oral health issues, the lack of data regarding access barriers to dental services is concerning. Therefore, this study investigates the barriers and facilitators Karen parents encounter when accessing oral healthcare for their children in Australia. This study will add further knowledge and deeper insights into refugee parents' experiences and opinions regarding their children's oral healthcare, which is essential for improving their oral health through tailored oral health promotion programmes.

## 2 Methods

### 2.1 Study Design

We adopted a qualitative research design employing the phenomenological approach, which prioritises understanding individuals' lived experiences and the interpretations they derive from them [27]. This methodology captures and appreciates firsthand experiences within their unique socio‐cultural settings [28]. This approach enables an objective and comprehensive exploration of refugee parents' or caregivers' perspectives regarding their encounters with oral healthcare in Australia. One‐on‐one in‐depth interviews were employed as the primary method, facilitating researchers in understanding participants' viewpoints, elucidating the importance of their experiences and exploring their lived realities before scientific interpretations [29, 30]. We chose semi‐structured interviews because they allow the researchers to use a standardised set of questions across all participants while permitting participants to provide spontaneous detailed responses [31]. Ethics approval was obtained from the La Trobe University Human Research Ethics Committee [HEC23186].

### 2.2 Theoretical Framework

The Penchansky and Thomas's model of access [32] and the modified Penchansky and Thomas's model of access by Saurman [33] served as our theoretical frameworks. According to Penchansky and Thomas's model, access refers to the alignment between the consumer and the service, with a better fit leading to improved access. It portrays access as a dynamic interface between patients and the healthcare system, shaped by several key dimensions: affordability, availability, accessibility, accommodation and acceptability. They are distinct yet interconnected, and each plays a crucial role in assessing the attainment of access to dental care. In 2016, Saurman introduced the concept of ‘awareness’ as a sixth dimension within the theory of access [33]. To guide our semi‐structured interviews effectively, we developed a topic guide (Table S1) aligned with the key dimensions to explore the barriers and facilitators experienced by parents when accessing oral healthcare for their children in Australia.

### 2.3 Participant Recruitment and Data Collection

We employed purposive sampling in this study to select individuals who exemplify the studied social phenomenon. We chose parents or primary caregivers (will be presented as parents henceforth in this article) aged 18 years and older, with at least one child under 12 years old, and who have used dental services. To ensure a diverse perspective, we recruited parents who were recent settlers (< 1 year) to those who have settled here several years ago (> 15 years). We aimed to interview 25 Karen refugee parents, continuing until data saturation was achieved. Saturation is reached when parents offer similar insights, enhancing the understanding of the phenomenon under study and when minimal to no new information emerges [34]. Recruitment was facilitated in collaboration with two non‐profit organisations, namely the Karen Organisation of Bendigo (KOB) and Bendigo Community Health Services (BCHS), who assisted in reaching out to potential parents. Information about the study, provided in both English and Karen languages, along with a consent form outlining the study's approach, was given to interested individuals. Those interested were invited to one‐on‐one interviews about their oral healthcare experiences.

Interviews took place between November 2023 and February 2024, in person, with the assistance of a professional interpreter from the Karen refugee community. Each interview, conducted at the parents’ preferred location, lasted between 35 and 60 min, including interpretation time. Before each interview, participant information and consent were addressed to ensure voluntary participation. All parents received a $40 gift voucher and an oral hygiene kit as an incentive. All interviews were audio recorded using an Olympus digital recorder, translated from Karen and transcribed into English. Transcriptions were conducted after every three to four interviews. This method improves accuracy, enables iterative refinement and allows for necessary adjustments to the interview process. The primary researcher (SBB), a male dentist with a master's degree and 9 years of research experience, conducted all interviews.

### 2.4 Data Analysis

We employed an inductive, data‐driven approach to analyse the interview data, focusing on open coding and emphasising participant‐based meanings. The generated codes purely reflected the data content without any preconceived theories or frameworks [35]. We followed Braun and Clark's six‐phase process for thematic analysis, which includes (1) familiarising with the data, (2) generating initial codes, (3) developing themes, (4) reviewing potential themes, (5) defining and naming themes and (6) producing the final report [36]. The primary researcher performed line‐by‐line coding through multiple readings, note‐taking and repeated transcript analysis over 4 months. This iterative process aimed to merge codes with similar meanings into themes or sub‐themes [37]. Major themes related to barriers and facilitators for accessing dental services and refugee parents' experiences with the oral healthcare system were identified. These themes were compared and discussed within the research team. The second and third researchers also reviewed the transcripts and themes. Selected quotations, identified by anonymised participant numbers and gender, were used to illustrate key points. In this article, parents adopted to access oral healthcare and a flowchart explaining their experiences with using oral healthcare in Australia. NVivo 14 Windows software (QSR International Pty Ltd.) assisted in coding and analysing the data [38]. This study is reported in line with the COREQ (COnsolidated criteria for REporting Qualitative research) checklist to facilitate transparency in reporting of qualitative research (Table S2) [39].

## 3 Results

### 3.1 Sample Characteristics

Twenty‐three parents, comprising seventeen females and six males aged between 24 and 52 years, were purposively recruited. All parents were interviewed face to face. Seventeen parents were recruited through KOB, while the remaining six through BCHS. The purposive sampling ensured participants had varied durations ( < 1 year to > 15 years) of resettlement and experiences with Australia's oral healthcare system. All interviews were conducted with the help of a professional interpreter who was from the same refugee community, except for three parents who were fluent in English. All parents had children aged 12 years or younger. The characteristics of the parents are presented in Table 1.

**Table 1:** Sample demographics of the Karen Refugee Parents (*n* = 23).

Participant code	Mother/Father	Age	Total no. of children/No. of children under 12 years	Years since their resettlement
P1	Mother	49	5/2	6
P2	Father	44	4/2	7
P3	Mother	41	4/4	11
P4	Mother	27	2/1	12
P5	Mother	47	4/1	14
P6	Mother	32	2/2	7
P7	Father	48	4/2	6
P8	Mother	28	2/2	17
P9	Mother	31	2/2	8
P10	Mother	33	2/1	15
P11	Mother	43	4/1	6
P12	Mother	29	2/1	17
P13	Mother	30	2/2	6
P14	Father	34	1/1	2
P15	Father	46	4/4	2
P16	Mother	24	3/3	15
P17	Mother	30	2/2	8
P18	Mother	52	2/1	1
P19	Mother	43	2/1	2
P20	Father	39	2/1	1
P21	Mother	37	4/1	1
P22	Mother	28	4/1	2
P23	Father	46	4/1	1

### 3.2 Themes

**Figure 1:**
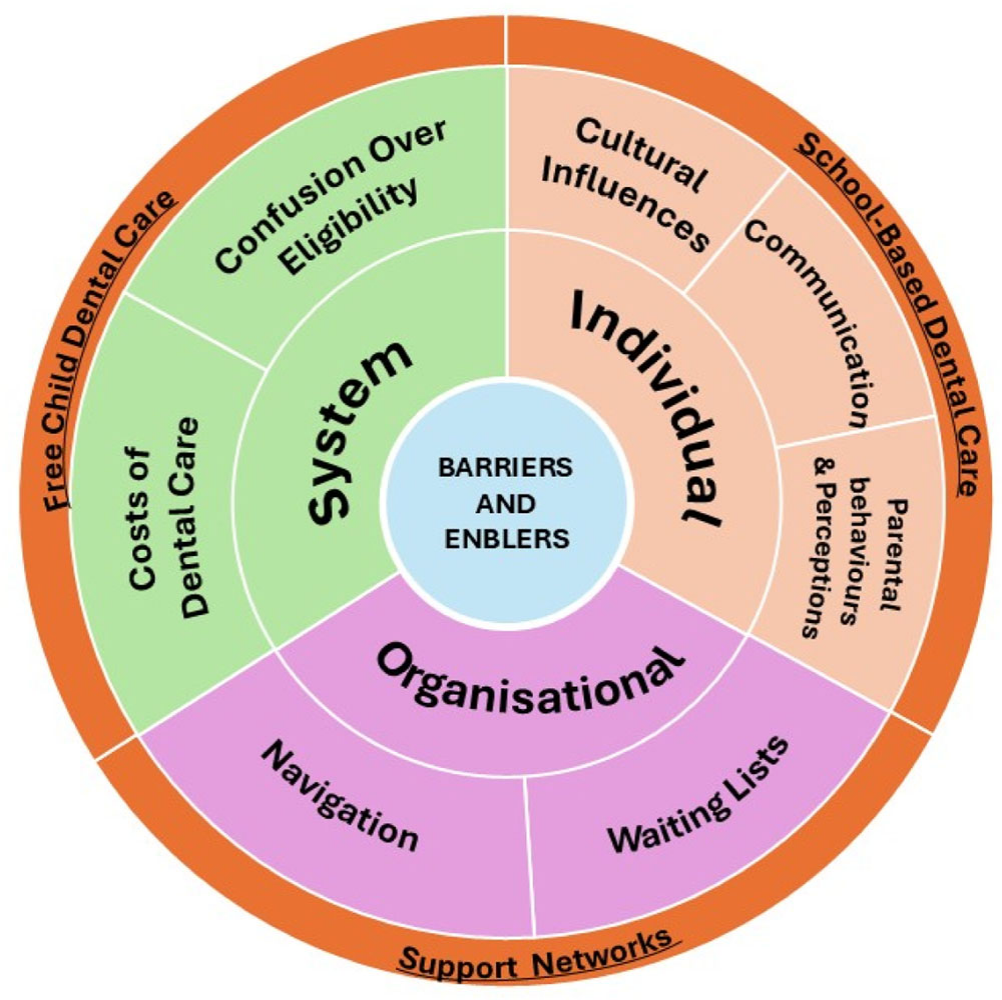
Barriers and facilitators to access dental services in Australia. Barriers to access appear in the inner and middle wheels, while the facilitators are presented in the outer wheel (underlined).

Nine main linked themes were identified from the analysis, which were reorganised under three categories derived from those themes: barriers to accessing care, facilitators to accessing care and the parents' experiences with dental care visits for their children. Figure 1 depicts the pictorial representation of the themes under the barriers and facilitators.

### 3.3 Barriers

The major themes concerning barriers were organised under three categories: individual, organisational and structural/system (Figure 1). More examples of the parent quotes to the themes are presented in Table S3.

#### 3.3.1 Individual‐Level

Cultural practices, parental behaviours and their perceptions emerged as the foremost barrier at the individual level (specifically identified by 17 parents). Some parents mentioned that they frequently handle their children's dental problems on their own. They felt confident using traditional remedies or pain‐relieving medications to treat these problems. They sought professional dental care only when the pain was severe and not alleviated by these methods. One participant mentioned as follows.

One of my children experienced gum swelling. I got him to use salt water and a slice of onion on the gum. I also gave him Panadol to take, and it took one or 2 days for the swelling to go away.

(P18, 52‐year‐old mother)

Parental lack of comprehension was seen as a key element for their behaviour. Most parents viewed baby teeth as having little value due to their temporary nature. They reported paying less attention to protecting baby teeth. In one interview, a 28‐year‐old mother shared her experiences with other parents from the Karen refugee community, and she described the parental attitudes towards child teeth as follows.

The parents of other children do not talk much. They do not care. They do not express. I encouraged that, oh, you should go get, um, a dentist checkup for your kids. They replied that they do not have any issue with their children's teeth because they are still in, um, they still have baby teeth. And that is not serious. They say…we will go see the dentist… when they have adult teeth.

(P8, 28‐year‐old mother)

Parents discussed their barriers with communication impacting access to dental care. Booking an appointment was identified as difficult, with limited English proficiency. Fourteen parents recognised this as a specific barrier. Similarly, two parents reported hesitancy in asking for the help of others to make appointments.

It was quite difficult for me to make an appointment with the dentist because if I had to make it myself,… I would not be able to do it. I do not speak English; it would be very difficult. Having to ask someone on my behalf to make the appointment is also not easy for me because sometimes I feel a bit hesitant to ask for help.

(P19, 43‐year‐old mother)

The need identified here is to create a supportive environment for seeking assistance, which can improve access to dental services for refugees with limited English proficiency. One participant emphasised the importance of persistent efforts in overcoming communication challenges, as scheduling appointments and communication are the barriers while this specific participant has oral health knowledge – whether she has time to phone the clinic again remains unknown: ‘stressing the importance of maintaining children's dental health’.

I tried it once… but I found it very challenging because I had to wait for a long time on the phone, and no one was answering the phone when I tried it. I did not try after that, but I plan to try again because the kids are growing up, and with not having good teeth, it is important to have good teeth, and I will try that again.

(P21, 37‐year‐old mother)

#### 3.3.2 Organisational/Clinic‐Level

At the clinical level, many parents were unhappy with the long waiting lists to see a dental service provider. Few parents mentioned negative experiences they faced when they tried to access dental care, evident from the following comments:

a staff from settlement service organisation made a call to the public dental clinic first, but there was a full booking or no spot available that we could get sooner, and my son's situation was worse and worse… so she booked my son with the private as we thought it might be quicker, but they did not do it for my son, so she called the public dental again…. it was not easy for me.

(P13, 30‐year‐old mother)

I don't like seeing the dentist, and for making an appointment, there is a 2‐week waiting list. I am not sure about other who has a problem with their teeth and how they do it. But for me last time, I had a serious tooth problem, and I contacted the dental services, and I was told that they couldn't make an appointment any soon… so, I went there myself, and they told me there was no available spot for me and they told me to go to another dental service.

(P12, 29‐year‐old mother)

One participant, a 28‐year‐old mother, shared her experience with a situation that made it very hard for her to get the help she needed for her child's dental care. She was stuck with a difficult choice: either wait a long time for public dental care or go to a private dental practitioner without an interpreter. She illustrated her experience as follows:

I think there will be a barrier if you don't speak English because if the private dentist takes you in, they need to book an interpreter, which will come out of their pocket. I don't think they want to do that. But then, if you wait for the government one, you'll have to go on a long waiting list. So, you either wait on the waiting list or see a private dentist without an interpreter when you really need it.

(P8, 28‐year‐old mother)

#### 3.3.3 Structural/System‐Level

Affordability was identified as a barrier to accessing oral healthcare. Several parents mentioned that they would delay or were reluctant to visit the dental practitioner because of these high costs.

I don't know. That's what I wanted to ask. According to what I heard from others, they say as an adult, there is a cost that we have to pay if we see the dentist, but I have no idea how much it costs. Because of the cost, I defer or am not willing to see the dentist.

(P3, 41‐year‐old mother)

When asked if cost was a barrier to their children's dental care or if they had delayed or deferred it, most parents acknowledged being aware of government support for children's dental services. However, they were unclear on the specifics of this funding. Many parents were unsure of the source of the support, while a few believed it came from Medicare or a concession card (Centrelink).

Some parents expressed concerns over the insufficient details regarding funding support and its limits. They argued that a lack of awareness of the financial benefits and information on government support would restrict the refugees from seeking dental care. This is because some refugee households have more children, or in some instances, the parents need to take their children for multiple dental visits.

the cost is a major barrier for me… if I have to pay on my own because I have four children, and none of them have good oral health. So, if I have to access dental services and need to pay for that, it would be a problem… I do not have any information on the support.

(P21, 37‐year‐old mother)

One participant described her frustration with the lack of clear information about dental care costs. Although she received a letter from the government indicating her child's eligibility for free dental care, she found it frustrating that the eligibility was not fully confirmed either in the letter or by the clinic administration. She commented as follows:

Coming to dental care cost, how much is the cost for a session? How much does filling cost? And the government said you may be eligible. That's whether I am going to be eligible or am I not going to be eligible? “Because the letter says, “you may be eligible.” Yeah, it does not say you are eligible. You may be… You may be… It is a big doubt and frustrating.

(P8, 28‐year‐old mother)

### 3.4 Facilitators

The findings under facilitators are reported under three major themes: support networks, free child dental care and school‐based dental care (Figure 1).

#### 3.4.1 Support Networks

Parents described the support they received from settlement service organisation and how it helped them access dental care in their initial days of resettlement. Parents also discussed the support from the settlement service organisation when they need to make a dental appointment. They spoke very positively about this support.

For me, when we moved here (Bendigo), we received support from settlement service organisation, and they provided us with information about dental checkups and booked dental appointments for my family.

(P4, 27‐year‐old mother)

It was easy to find a dentist in Bendigo, but I did not go to the dentist to book an appointment. I am new in Bendigo; I rely on the community at settlement service organisation…. If I have any concerns with teeth and have to make an appointment, I will get a BCHS worker to assist me in doing that.

(P23, 46‐year‐old father)

When asked, ‘Did you try other sources or reach out to anyone when you needed to consult a dental practitioner?’ most parents indicated that they sought assistance from intermediaries, such as friends, family members, general practitioners, interpreters, school teachers, or community leaders like pastors.

*Y*es. Whenever settlement service organisation staff are unavailable, I ask support from the pastor, and he will arrange times and sort things out for the family.

(P18, 52‐year‐old mother)

There was once when I took my older son to the GP and asked if he could organise the referral to see the dentist at the dental service. The GP arranged a dental appointment for my son and told me there are some Karen dental assistants working there as well.

(P6, 32‐year‐old mother)

**Figure 2:**
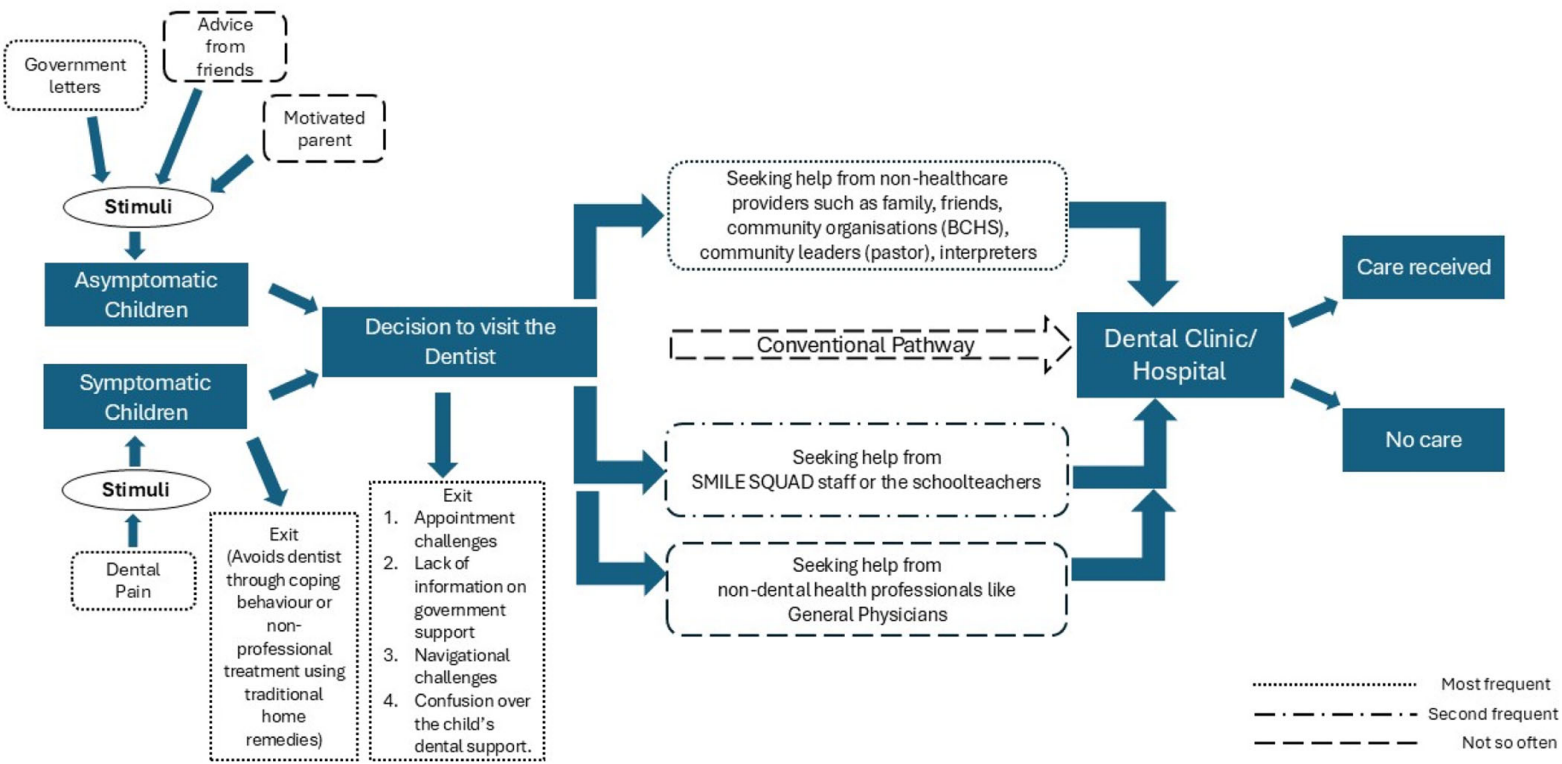
Dental care pathways for children from Karen Refugee Community.

This illustrates the proactive efforts and resourcefulness of refugee parents in seeking oral healthcare for their children despite facing significant barriers through leveraging support from various communities and healthcare networks. Figure 2 shows the dental care pathway model Karen refugee parents often use to consult a dental service provider for their children.

#### 3.4.2 Free Child Dental Care

While some parents were unaware of government support for children's dental care, a few acknowledged their awareness of this support. The parents who obtained free dental care for their children from public dental clinics were thankful for the opportunity. They shared that it has significantly helped improve their children's oral health when needed.

When we first arrived here, an appointment was arranged, and there was a budget that was under a government program. When we visited the dentist, money was deducted from the budget under that program. When we received the dental appointment letter and went to see the dentist, we were explained how much money was left or remained so that we could come and see the dentist again. We have been to the dentist several times and were never asked to pay. We were happy with that support.

(P2, 44‐year‐old father)

When we arrived here and attended the oral health education, they wrote to us with some support, and that's when I got to know that the dental services are free for children.

(P21, 37‐year‐old mother)

Parents mentioned the anxiety and uncertainty faced by newly arrived refugees regarding the cost of dental care over time. Despite being aware of the CDBS, there is also fear that, as time passes, they may incur fees. One participant commented as follows.

I do not have any concerns at this stage. The kids got the checkup done at school, and the examiner said that there was no problem, no major problem at all. But as I am newly arrived here, I wanted to get the kids’ checkup done because I heard it is free. But I worry that if I am here for longer, later, if I go to the dentist…. I will have to pay. That's what I heard.

(P22, 28‐year‐old mother)

#### 3.4.3 School‐Based Dental Care

Many parents reported that they were aware of school‐based dental care for their children and how these dental visits at school helped keep their children's oral health in check. The school programme also taught parents the importance of oral healthcare in younger age groups and when to seek support for their children. For example, one parent shared how they rely on school dental services to make a dental appointment with the public dental care provider.

There is an oral health program that comes to my children's school every year. So, my children brought some forms home for us to complete them. If we want to get them to see the dentist, we complete the forms and receive the appointment letters soon after from Bendigo health dentist service. My children brought some goodies for dental care, like a water bottle with tooth symbols each time the oral health program. So, I always wait for an update from school for dental appointments for my children…. I do not go and make appointments myself.

(P3, 41‐year‐old mother)

Another participant discussed how the school‐based dental programmes educated their children in practicing proper oral hygiene and empowered them to maintain their oral health effectively.

When my daughter goes to school, there is a dental program coming to her school. The dentist provided checkups and asked her how we must brush our teeth. My daughter said we must brush our teeth when we wake up, and the health staff told her that… no, that is not right, we must clean it after we eat, like after breakfast, and other one is before going to bed.

(P4, 27‐year‐old mother)

### 3.5 Experiences

**Figure 3:**
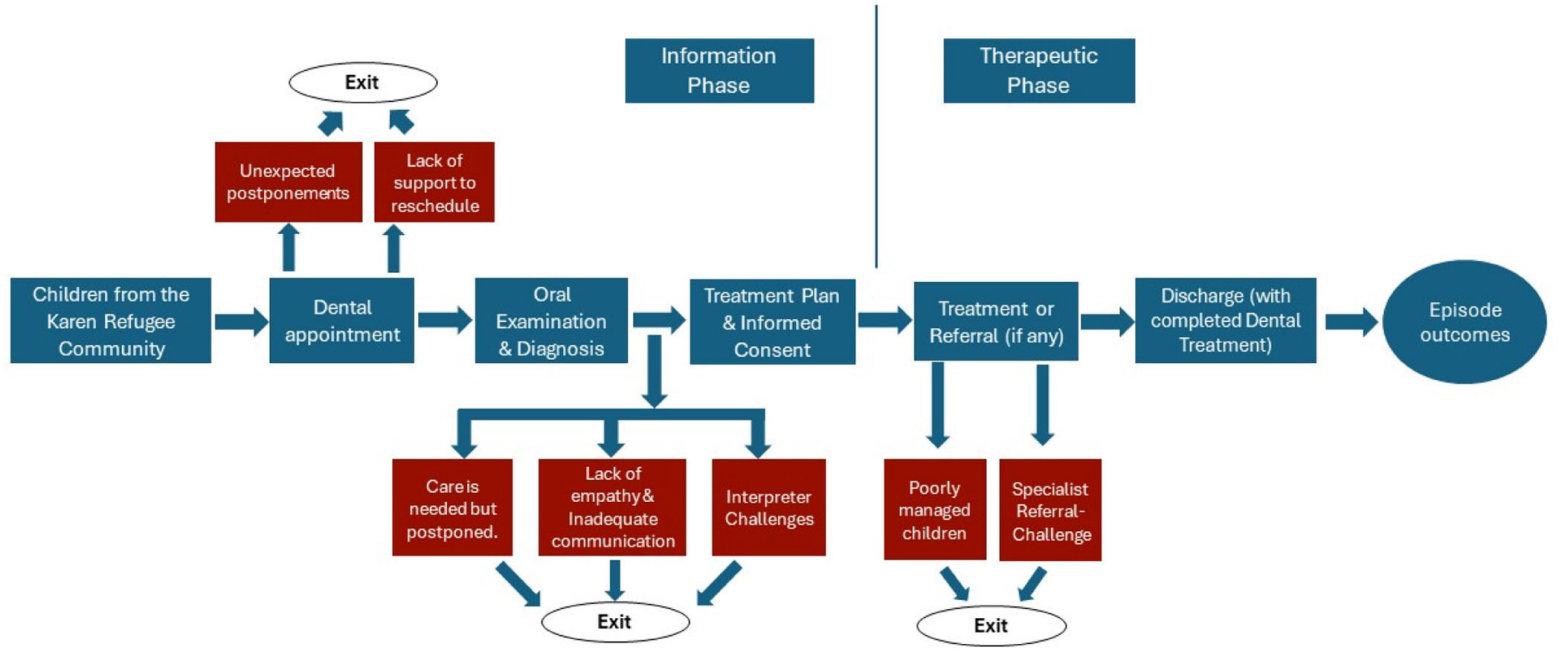
Dental Care Episode showing the parents' experiences. Red boxes in the flowchart denote the negative experiences of parents with the dental service providers, interpreters and supporting clinical staff.

The findings under experiences are reported under three major categories based on their interactions with dental service providers, interpreters and supporting clinical and administrative staff. Figure 3 illustrates the dental care episode model depicting the parents' experiences when they took their children to the dental service providers and their influence on oral healthcare utilisation.

#### 3.5.1 Satisfaction

Many parents expressed satisfaction with their interactions with dental service providers. They appreciated the detailed explanations of treatment procedures, advice on personal oral hygiene and treatment possibilities and effective communication methods, such as pictorial factsheets, factsheets in Karen and body language. On the other hand, parents also recounted their experiences with dental service providers, which led them to decide against returning to these providers for children's dental care.

The first reason why I do not want to go see a dentist is.. each time when I take my daughter, I do not see the same dentist…. each time, different dentist, some are gentle and friendly…. while others, they are not like…. friendly.. when they speak, I feel like they didn't want to talk with us… I do not know from their side; from my side, I feel like I do not belong.. they are not really happy, and they are not quite friendly. I feel like they don't want to speak with me; they speak very low.. it is very hard to hear that… also, they handled the children roughly or strongly, so for me, it is not okay.

(P12, 29‐year‐old mother)

Parents generally found comfort in having interpretation services available during consultations, which helped them understand advice and treatment. However, not all parents were completely satisfied with the interpreters; some encountered issues such as dialect differences in phone interpretation, the lack of continuous and reliable interpretation services, and the limited interpreter availability.

When I attended the dental appointment, they did not provide an interpreter, so I took one of my nephews with me.

(P18, 52‐year‐old mother)

At that time, there was a lack of interpreters… there were only phone interpreters… and they provided me with one. I felt difficulty with the interpreter at that time because the dialects that I speak are different from the ones that the interpreter spoke, and we did not understand each other.

(P12, 29‐year‐old mother)

Parents generally reported positive experiences with the administrative staff, finding their interactions satisfactory. The staff were friendly, greeted parents warmly, showed respect, and provided clear guidance during consultations. Few parents acknowledged how happy and comfortable they were with the presence of Karen dental assistants within the clinic as supporting staff.

I thought it would be best to see a familiar face because I know that a lot of Karen work there at the public dental clinic. As a dentist assistant, many Karen young women work there…. they are very supportive.

(P8, 28‐year‐old mother)

#### 3.5.2 Accessing Specialist Care

Few parents described their challenges when local dental services could not offer the required treatments, necessitating referrals to specialists in distant cities like Melbourne. They emphasised the difficulties of navigating unfamiliar territory without sufficient assistance, especially as newcomers. This underscores the call for improved local dental care options and stronger support systems to help families access specialised care when needed.

I was told by the dentist in Bendigo that they cannot do the treatments here in Bendigo. The only way is to send the children to a specialist in Melbourne… The dentist sent him (my son) to Melbourne, and it was quite hard for me to navigate the way there. I did not receive support from BCHS because the BCHS staff was unavailable then. So, I asked my relative to take us there. It was challenging because I was new here and had never been to Melbourne. I felt a bit upset during the time.

(P18, 52‐year‐old mother)

#### 3.5.3 Dental Appointments

Some parents expressed dissatisfaction with their experiences, citing unexpected postponements of dental appointments, frustrations with communication barriers with reception staff, and a perceived lack of support when trying to reschedule appointments.

The experience with the dentist was good. He is friendly. However, during that time, he (kid) had a knee injury because he had a fall, and I wanted to reschedule the appointment for next time because he was experiencing pain in his knees as well, but I was not comfortable enough to ask to reschedule. I raise the question to the administrative staff… I did not get a reply, and I wasn't sure if I had the right to ask or not for rescheduling.

(p23, 46‐year‐old father)

### 3.6 Improving Oral Health Knowledge and Access to Dental Care

We specifically sought suggestions from the parents on how to improve their children's oral health and enhance access to dental care. The majority recommended organising group information and education sessions to convey oral health information. A few parents suggested a combination of methods, including educational videos in their native language, group sessions and factsheets, while some recommended cross‐cultural education to dental service providers. They quoted as follows:

Having an information session would be the best option for me because everyone can come… sit there… get the information delivered to us, and we will also be able to discuss as well.

(P18, 52‐year‐old mother)

It is a combination for me. Coming to the session in person and also having the video as well and then at the end provide the fact sheet so that whenever I need to refer back, I can always go back to the fact sheet to remind myself. So, it is a combination of all materials.

(P20, 39‐year‐old father)

## 4 Discussion

This study addresses a gap in the literature by examining the barriers and facilitators to accessing dental services, highlighting the firsthand experiences of Karen refugee parents regarding their children's oral healthcare in Australia's multicultural context. The study's findings reveal that Karen refugees encounter numerous challenges in accessing dental care. These challenges align with existing literature, which identifies cultural practices, limited understanding of the healthcare system, language barriers, inadequate interpretation services and dental care costs as significant barriers hindering refugees' access to dental services [40, 41]. Additionally, they described limitations in their capacity that hindered their social participation, including accessing dental care. Also, external factors beyond the parents' control were the primary influences on their dental care experiences. Almost one‐third of the parents had been in Australia for less than 5 years, with most having been here for only one to 2 years. Their limited understanding of how the dental care system works further contributed to delays in accessing dental services.

In this paper, we utilised our study data to develop a model illustrating the dental care pathway based on the personal experiences of Karen refugee parents in accessing dental care for their children in Australia. The most frequent pathway identified was through non‐healthcare providers, followed by dental service providers from the SMILE SQUAD programme and school teachers. Smile Squad is a Victorian government programme offering free dental care to students in public primary and secondary schools. It focuses on improving children's oral health through preventive services like check‐ups, cleanings, fluoride treatments and necessary restorative care. Less common pathways included the conventional route and those involving non‐dental health professionals. These pathways align with the ‘lay referral system’ described by Grembowski et al. [42]. According to this framework, low‐income individuals often resort to personal coping mechanisms for dental issues. Similarly, our study found that refugee parents frequently used traditional home remedies, highlighting cultural practices as a barrier to oral healthcare utilisation. Our model resembles those developed for humanitarian migrants in Canada, except for transnational care, where individuals sought dental treatment and medications from outside their country of residence [43, 44]. Furthermore, our dental care episode model revealed various phases of negative experiences in dental care, indicating that the dental needs of Karen refugee children are not being adequately met in Australia, leading parents to delay seeking oral healthcare for their children.

Any dental or medical care system aims to provide services to those in need. In Australia, refugees have been shown to demonstrate higher rates of dental diseases compared to the general population [45, 46]. However, access to dental care follows the inverse care law; those who need the care are the most often not received, indicating that inequitable access to dental care continues to be a major public health issue [42, 47]. Despite introducing policies that provide priority access to public dental services to the refugee populations, it seems to have a negligible effect on dental care utilisation among this population. The 2016 audit suggests limited participation rates among refugees, approximately 17% [48]. In short, other factors, such as behavioural, cultural, provider and service delivery systems, appeared to be important factors in determining accessing dental services by priority groups.

The main theme concerning the barriers to accessing oral healthcare, as described by the interviewees, is rooted in cultural beliefs that prevent them from seeking dental care. Parents often mentioned that they would seek dental help only for visible issues like tooth decay, with pain being the main reason for seeking care, similar to findings in other ethnic groups [49]. Many interviewees generally did not prioritise dental care for their children, as they still had baby teeth. This indicates that their beliefs, practices and knowledge about oral health served as barriers to seeking dental care. The lack of motivation for routine dental visits, reliance on traditional home remedies, and low help‐seeking behaviour emerged as a significant barrier. The strong influence of cultural stereotypes about oral healthcare leads to delayed dental care. Therefore, recognising the diversity among Karen refugees and implementing cultural competence in oral healthcare services is crucial.

Parents had varied experiences with free dental care for children. For Karen refugees, dental coverage through the CDBS was a relief because they did not have to pay for their children's dental treatment. However, many expressed concerns about the lack of detailed information on this coverage. Some parents were dissatisfied when they received letters stating their children ‘may be’ eligible for government support through CDBS. A few parents were unclear whether the CDBS applied to their family with more than three children or if they could use it for multiple dental visits. This confusion led some refugees to mistakenly believe they were not eligible for CDBS benefits, which delayed oral healthcare utilisation. This inconsistent information on their children's eligibility for free dental care, wherein the parent was left with confusion regarding seeking dental care, should be replaced by a more efficient public dental care system with clear, consistent information about dental care benefits and costs to alleviate concerns and ensure that new arrivals can confidently access necessary dental services for their children without fear of unexpected expenses.

To address common barriers like long waiting times and financial constraints, the Department of Health and Human Services of Victoria implemented new policies that prioritise refugees for immediate appointments and exempt them from service fees [20]. Despite these measures, many parents in this study were dejected by the long waiting lists in the public dental system. They described being on waiting lists and waiting for dental appointments for their children, causing anxiety that delays could worsen their children's oral health issues. Some parents also discussed the challenges of choosing between public and private dental care. Waiting for public services could mean treatment delays, while private care might involve language barriers and additional costs. These experiences underscore the need for effective solutions to shorten wait times and enhance access to affordable dental care, particularly in private settings.

Efficient service provision involves allocating and using resources optimally to achieve desired outcomes [50]. Empathy is crucial when providing care for refugees and humanitarian migrants, who often face stigma and misunderstandings [51]. The study indicated parents valued being involved in treatment planning decisions. However, some reported brief interactions with dental service providers and felt that communication with them was lacking. Despite this, they attributed the issues to the system rather than the staff, acknowledging that healthcare professionals must balance patient needs with available resources and regulatory limits. Interview data indicated that language barriers were a significant issue during dental care, primarily due to the lack of interpreters throughout treatment. This communication breakdown negatively impacted the dental care experience for some parents.

The parents suggested various recommendations to improve access to dental information, treatment and prevention. Most parents expressed satisfaction with the support provided by the Australian government. To enhance access to dental services, parents recommended oral health education sessions, either in groups or one‐on‐one, as beneficial. To address language barriers, they requested oral health information in the Karen language. A few parents also asked for educational videos in their native languages due to the variety of dialects within the Karen community. Additionally, they recommended cultural sensitivity training for dental service providers to improve their experiences during dental care visits.

### 4.1 Strengths and Limitations

To the best of our knowledge, this is the first study to explore the dental care experiences of parents from the Karen refugee community, focusing on their children's dental care and the barriers and facilitators they face in accessing dental services in Australia. The results are not meant to be generalised, as this is not the goal of this qualitative evaluation. The primary researcher's background as a dentist and immigrant might have helped parents feel more trusting, allowing them to speak freely about the issues in the dental system and reducing the usual power imbalance. After each interview, most parents verbally thanked the primary researcher for listening to them and appreciated his efforts in improving their community's oral health. They also expressed interest in learning more about oral healthcare by participating in future stages of the research project.

A limitation of the study is the small number of male parents. This was due to difficulties encouraging participation, mainly because of work schedules and limited time. Additionally, the recruitment efforts by KOB and BCHS primarily attracted female parents, which likely led to further female participation. Another potential limitation is that parents may have reported more favourable oral health behaviours due to social desirability bias or the fact that the facilitator was a dental practitioner. Furthermore, the study's sample consisted solely of parents from one refugee community. Thus, the results are based on subjective perceptions and should not be generalised to parents from other refugee backgrounds.

## 5 Conclusion

Understanding why dental services are underutilised in refugee communities, especially for children, is crucial for promoting health equity. Our study findings revealed lower utilisation of dental services within the Karen refugee community, and their experiences highlighted gaps in the provision of oral care services. Cultural beliefs and parents' views on oral healthcare as a low priority for children were major barriers to accessing dental care. Many parents cited a lack of knowledge about financial benefits, confusion about the level or amount of government support provided for children's dental care, and long waiting lists in the public dental system as reasons refugees might avoid seeking dental care. Although, our findings suggest that cultural practices posed as a barrier, making services more accessible, less confusing and better aligned with cultural practices, access to oral healthcare could potentially increase within this refugee community.

Improving the oral health of this refugee community requires multisectoral collaboration. To achieve this, stakeholders, including dental service providers and policymakers, must listen to the voices of the refugee population. Additionally, service providers are a key part of this environment as they can create positive experiences that increase the chances that these people adopt good dental habits. Therefore, listening to the experiences and challenges of dental service providers and interpreters who are integral to providing oral healthcare to these communities is crucial before designing tailored oral health promotion programmes.

## Author Contributions

**Sudheer Babu Balla:** conceptualisation, data curation, methodology, writing–original draft, writing–review and editing, software, investigation. **Jyothi Tadakamadla:** conceptualisation, supervision, writing–review and editing, formal analysis. **Santosh Kumar Tadakamadla:** conceptualisation, supervision, writing–review and editing, formal analysis.

## Ethics Statement

The study adhered to all applicable guidelines and regulations. Ethical approval was obtained from the La Trobe University Human Research Ethics (HEC23186). Participants provided voluntary informed consent, and their responses have been anonymized to ensure confidentiality.

## Conflicts of Interest

The authors declare no conflicts of interest.

## Supporting information

Supporting information.

## Data Availability

Due to the nature of the research, supporting data are not available for ethical reasons. The participants of this study did not give written consent for their data to be shared publicly.
