# Supplementary material for: ‘The Letter Says I May or May Not Be Eligible… It Is a Big Doubt and Frustrating:’ A Qualitative Study on Barriers and Facilitators to Children's Oral Healthcare From the Perspective of Karen Refugee Parents in Victoria
Source: Health Expect. 2024 Nov 19;27(6):e70110. doi: 10.1111/hex.70110 (PMC11576330; doi:10.1111/hex.70110)
Supplement: Supplementary file 1 — Supporting information. [file HEX-27-e70110-s001.docx]

**Supplementary Table 1:**

**Interview Topic Guide- Questions for Parents of children (<12 years) of the Karen refugee community in the regional Victoria**

**Section 1: Sample Demographic**

1. **Can you please tell me about yourself, your family, or the people you live with?**

**Prompts for further information:**

- Whom do you live with?
- How many children do you have?
- How old are they?
- What is the educational background of you and your spouse?
- When did you move to Australia?
- For how many years are you staying in Bendigo?

**Section 2: Oral Health Beliefs, Quality of life, and well-being**

1. What is good oral health according to you?
2. Where did you learn about dental care?
3. When do you think or say that your child has “good oral health?”
4. How would you describe a child with good oral health?
5. What could be the reason for poor oral health?
6. Are dental problems as serious as other health problems?

**Prompts for the oral health of their children:**

- How do you feel about the ultimate oral health of your children?
- How concerned are you about your son or daughter developing dental problems (dental decay)?
- Does any of your friend’s children in your community have dental problems? How common is it among children?

**Section 3: Role of parents in the oral health of their children**

1. What do you think parents can do (or you as a parent can do) to prevent dental problems in kids (your kids)?
2. As a parent, what do you think are the most important ways to help a child have good oral health?

Prompts for the parental knowledge:

- What activities can you do to improve your child’s oral health?
- What about diet and oral health?
- What about fluoride and oral health?

**Section 4: Previous use of dental services**

1. Have you accessed dental services in Australia?

| **If YES:** | **If NO:** |
| --- | --- |
| 1. How often (e.g., per year) do you (and your family) access dental health services? ****Prompt for children**** | For what reasons have you not accessed dental services? |
| 1. How does this compare to your use of other services? | Prompts for psychological factors:   - How do you feel about seeing a dentist? - In your home country, is it common for people to use dentists? - Do you think it is different seeing a dentist here compared to other countries?   Prompts for practical factors:   - Do you know about dental services that are available to you? - Do you feel that you are able to get to dental appointments? - Do you think that dental services are affordable? |
| 1. Who within your family or close social circle used dental services? Is it everyone or only some members, and why? |  |
| 1. For what reasons have you (and your family) used dental services? (i.e., regular check-ups, critical situations) |  |

1. Have you accessed dental services before coming to Australia?

| **If YES:** | **If NO:** |
| --- | --- |
| 1. How often did you (and your family) access dental services? ****Prompt for children**** | For what reasons have you not accessed dental services? |
| 1. How does this compare to your use of other services? | Prompts for psychological factors:   - How did you feel about dental services in your home country?   Prompts for practical factors:   - How easy was it to access dentists? - Did you know about dental services that are available to you? - Were dental services affordable? |
| 1. Who within your family or close social circle used dental services? Is it everyone or only some members, and why? |  |
| 1. For what reasons have you (and your family) used dental services? (i.e., regular check-ups, critical situations) |  |

**Section 5: Help-seeking**

1. How important do you think it is to go to a dentist?

- How important do you think it is compared to other healthcare services?

1. How easy or hard do you find it to go to the dentist? (why do you think that is?)

- How about compared to others?

1. Did you receive any support from your friends or your community who helped you to get dental care for your children?

**Section 6: Experiences with dentists and dental services**

1. Can you tell me about your experiences with healthcare services in Australia?
2. Can you tell me about your experiences with dental services in Australia?

- Did you see a dentist on arrival in Australia? Does anyone help you with registering for a dental service? If yes, how did you find that experience?
- How did the dentist make you feel? (comfortable? Welcome? Cared for?)
- Did you ever feel that communication between you and your dentist is not appropriate?
- Did you ever feel that you were not treated properly?
- Are the staff at the dental service friendly? Did they provide you with an interpreter to understand your or your child’s dental problem?
- What other things made your experience more positive or negative?

1. Cost of the dental services

- Do you feel protected financially against possible expenses for your child’s dental care?
- Did your child not get the recommended dental treatment because of the cost?
- Did you defer or delay your child’s dental visit because of the cost?
- Did the dental service ever keep you on the waiting list even though you need emergency treatment?

1. What are other barriers you think of to accessing dental care when needed?

- Is the dental service within reach, or do you have any trouble with the transport?
- Does working days/ hours of clinic prevent you from getting dental care when needed?

1. What would make you want to go back to see that dentist?

- What does appropriate care mean to you?

**Section 7: Recommendation**

1. What would you recommend for dentists to provide better care for your children and others in your community?
2. How important is educating the parents or caregivers on the importance of oral health?
3. Are you aware of any school or community-based dental programs for children?

- Do you think these programs are important?

**Supplementary Table 2: COREQ checklist**

**Consolidated criteria for reporting qualitative studies (COREQ): 32-item checklist**

| **Item No** | | **Guide Questions/Description** | **Reported on Page #** |  |
| --- | --- | --- | --- | --- |
| **Domain 1: Research team and reflexivity** | | | |  |
| **Personal Characteristics** | | | |  |
| 1. Interviewer/ facilitator | | Which author/s conducted the interview or focus group? | Pg 4 |  |
| 2. Credentials | | What were the researcher’s credentials? E.g., PhD, MD | Pg 4 |  |
| 3. Occupation | | What was their occupation at the time of the study? | Pg 4 |  |
| 4. Gender | | Was the researcher male or female? | Pg 4 |  |
| 5. Experience and training | | What experience or training did the researcher have? | Pg 4 |  |
| **Relationship with participants** | | | |  |
| 6. Relationship established | | Was a relationship established prior to study commencement? | Pg 4 |  |
| 7. Participant knowledge of the interviewer | | What did the participants know about the researcher? e.g. personal goals, reasons for doing the research? | Pg 4 |  |
| 8. Interviewer characteristics | | What characteristics were reported about the interviewer/facilitator? e.g. Bias, assumptions, reasons and interests in the research topic | Pg 4 |  |
| **Domain 2: study design** | | |  |  |
| **Theoretical framework** | | |  |  |
| 9. Methodological orientation and Theory | What methodological orientation was stated to underpin the study? e.g. grounded theory, discourse analysis, ethnography, phenomenology, content analysis | Pg 3 |  |  |
| **Participant selection** | | |  |  |
| 10. Sampling | How were participants selected? e.g., purposive, convenience, consecutive, snowball | Pg 3 |  |  |
| 11. Method of approach | How were participants approached? e.g., face-to-face, telephone, mail, email | Pg 4 |  |  |
| 12. Sample size | How many participants were in the study? | Pg 5 |  |  |
| 13. Non-participation Setting | How many people refused to participate or dropped out? Reasons? | Pg 4 |  |  |
| 14. Setting of data collection | Where was the data collected? e.g., home, clinic, workplace | Pg 4 |  |  |
| 15. Presence of nonparticipants | Was anyone else present besides the participants and researchers? | N/A |  |  |
| 16. Description of sample | What are the important characteristics of the sample? e.g. demographic data, date | Pg 5 |  |  |
| **Data collection** | | |  | No |
| 17. Interview guide | Were questions, prompts, and guides provided by the authors? Was it pilot tested? | Pg 3 |  |  |
| 18. Repeat interviews | Were repeat interviews carried out? If yes, how many? | N/A |  |  |
| 19. Audio/visual recording | Did the research use audio or visual recording to collect the data? | Pg 4 |  |  |
| 20. Field notes | Were field notes made during and/or after the interview or focus group? | Pg 4 |  |  |
| 21. Duration | What was the duration of the interviews or focus group? | Pg 4 |  |  |
| 22. Data saturation | Was data saturation discussed? | Pg 4 |  |  |
| 23. Transcripts returned | Were transcripts returned to participants for comment and/or correction? | N/A |  |  |
| **Domain 3: analysis and findings** | | |  |  |
| **Data analysis** | | |  |  |
| 24. Number of data coders | How many data coders coded the data? | Pg 4 |  |  |
| 25. Description of the coding tree | Did the authors provide a description of the coding tree? | N/A |  |  |
| 26. Derivation of themes | Were themes identified in advance or derived from the data? | Pg 4 |  |  |
| 27. Software | What software, if applicable, was used to manage the data? | Pg 4 |  |  |
| 28. Participant checking | Did participants provide feedback on the findings? | N/A |  |  |
| **Reporting** | | |  |  |
| 29. Quotations presented | Were participant quotations presented to illustrate the themes/findings? Was each quotation identified? e.g., participant number | Pg 5-11 |  |  |
| 30. Data and findings consistent | Was there consistency between the data presented and the findings? | Pg 5-11 |  |  |
| 31. Clarity of major themes | Were major themes clearly presented in the findings? | Pg 5-11 |  |  |
| 32. Clarity of minor themes | Is there a description of diverse cases or a discussion of minor themes? | Pg 5-11 |  |  |

**Supplementary Table 3: Themes and sample quotes to the barriers and facilitators to access dental services in Australia and the refugee parents’ experiences with dental service providers for the oral healthcare of their children**

|  | Themes | Sub-theme | Sample Quotes |
| --- | --- | --- | --- |
| Barriers | Individual | Parental misconceptions about the importance of dental care for children | “*Because I know our teeth are very important and are part of our bones. But, some other parents, do not like taking their children to the dentist, and they said they do not take their children to the dentist frequently as there aren’t many changes. So, they do not take their children for regular check-ups. And yeah, I do not want to talk much, as people will say I am acting very high or bossing them around. In my opinion, those parents don’t understand about the oral health of their children.” p13, 30-year-old mother* |
|  | Organizational | Long waiting times in the public dental system | *“When I called the dental hospital and asked for an appointment for sooner spots, I was told to wait 2- 3 weeks. When I asked for a sooner appointment, they told me to visit a private dentist. For me, waiting for 2-3 weeks is difficult… I am happy and angry at the same time… it depends on the situation of the children.” p16, 24-year-old mother* |
|  | System | Confusion over the eligibility for the government scheme | *“When I enquired about a dental check-up for myself, I wanted to get my teeth done because I wanted to check my oral health. She (the receptionist) said because I'm not covered by the government scheme, I have to pay. It was probably $100 or 200. But this amount, you know, could be difficult for some people, you know, like to pay for that if they don't work or if they don’t have a high income, you know what I mean? It could be a problem. For myself, maybe I should do it for once.” p8, 28-year-old mother* |
|  |  |  | *“Those who have children and they have tooth problems, and if they have to take their children 4 or 5 times, will that scheme cover it? or if they have to pay, the dentist needs to explain to them clearly how much they have to pay. Otherwise, when it comes to paying from our own pocket, it would be difficult.” p12, 29-year-old mother* |
| Facilitators | Support networks | Support from the settlement services | *“It is mainly the settlement service organisation here. I have heard a few times about oral healthcare from my friends, but it was not much of a help. So, it was mainly the community here that supported me.” p20, 39-year-old father* |
|  |  | Support from interpreters to make dental appointments | *“This is how I make an appointment because I see my doctor at the Bendigo Primary Care, and after the appointment, I ask an interpreter to make an appointment for the dentist.” p3, 41-year-old mother* |
| Experiences | With dental service providers | Feeling neglected | *"There was one time with an emergency situation. My son had a front tooth problem with sore and swollen gum, and I took him to the private one as there was a waiting list at public dental. That female dentist was so funny because she assumed that my son fell and hurt himself, and I tried to explain it was not like that; my son seriously had tooth decay, but she kept arguing. I think she may be busy or impatient… also, my son was crying; she tried to settle him, but it did not work…she might be angry about that. So in the end, I had to take my son to Bendigo Health Dental Hospital, then they took it out.” p13, 30-year-old mother* |
|  | With Interpreters | Lack of adequate number of interpreters | *“What I want to bring up is the barrier of an interpreter. I would like to suggest that the services (dental) hire more interpreters because there was like when the interpreter was helping the dentist and client, it was not finished yet, but the time for the interpreter was almost finished, or another dentist came and asked for the interpreter as other patient had waited for so long already with a severe problem. So, even for the remaining time when the dentist and patient wanted to have more conversation, they could not be due to the language barrier, so I want to suggest getting more interpreters in the industry (p7, 48-year-old father).”* |
|  |  | Unpleasant experience during dental care episode due to lack of interpreter’s availability | *“I had an experience where there was an interpreter for the beginning of the appointment, and the procedure process was explained to me properly, including raising my hands if I felt pain so the dentist would stop. But during the procedure, there was no interpreter, and I felt pain. I put my hand up, but they didn’t stop, so they continued until the finish (p6, 32-year-old mother).”* |
